# MircroRNA-19a promotes vascular inflammation and foam cell formation by targeting *HBP-*1 in atherogenesis

**DOI:** 10.1038/s41598-017-12167-z

**Published:** 2017-09-21

**Authors:** Heming Chen, Xiaoyi Li, Shuiyi Liu, Lu Gu, Xinmin Zhou

**Affiliations:** 1 Department of Cardiovascular Surgery,The Second XiangYa Hospital, Central South University, Changsha,Hunan, 410011 China; 2 Department of Medical Laboratory, Central Hospital of Wuhan, Wuhan,Hubei, 430014 China

## Abstract

Atherosclerosis, a serious threat to human cardiovascular health, involves inflammation throughout its various stages of development. MicroRNAs play an important regulatory role in macrophages that respond to inflammation, but the underlying mechanisms are largely unknown. In this work, we study the impact of miR-19a in macrophage-derived foam cell formation during atherogenesis. A microarray-based analysis of serums from patients with coronary heart disease in comparison with healthy controls reveals a significant enrichment of miR-19a in the serums of atherosclerosis patients. A higher level of miR-19a is also observed in atherosclerosis-prone ascending aortic wall tissues than in internal mammary artery amongst patients with coronary heart disease. We identify *HMG-Box Transcription Factor 1* (*HBP-1*) as a target gene of miR-19a. HBP1 is repressor of macrophage migration inhibiting factor (MIF) and overexpression of miR-19a increases MIF expression. By administering a miR-19a antagonist to the caudal vein, we found a decrease in atherosclerotic plaques and lipids load in apoE-null mice fed with high-fat diet. These results support inhibition of miR-19a reduces inflammatory reaction and constitutes a potent therapeutic approach against atherosclerosis.

## Introduction

Atherosclerosis is responsible for several important adverse vascular diseases, including coronary artery disease, cerebral stroke, and peripheral artery disease, which account for most cardiovascular mortality and morbidity^1,2^. Atherosclerosis is a chronic inflammatory disease^3,4^, which is characterized by the accumulation of lipids and inflammatory cells in the vessel wall. Monocytes respond to chemotactic factors produced by vascular endothelia cells and smooth muscle cells and migrate from the peripheral blood into the arterial intima and differentiate into macrophages. At the same time, LDL (low density lipoproteins) are modified by reactive oxygen species (ROS), resulting in formation of oxLDL (oxidized LDL). oxLDL is an autoantigen^5–7^, which are taken up by macrophages to form foam cells-the hallmark of an early atherosclerotic lesion. MicroRNAs (miRNAs) are an abundant class of ~22nt endogenous noncoding single-stranded RNAs, which post-transcriptionally regulate gene expression by base pairing to imperfect complementary sites in the 3′ untranslated region of their target mRNAs, repressing their translation or promoting their degradation^8–11^. Recent studies show that miRNAs are involved in the pathogenesis and progression of atherosclerosis and are potential diagnostic or prognostic biomarkers and therapeutic targets^12–15^.

In this study, we demonstrated that miR-19a is highly abundant in the blood and tissue of patients with atherosclerotic coronary artery disease and identified HMG box-transcription protein1 (HBP1) as a new target for miR-19a. As a transcriptional repressor^16^, HBP1 participates in inhibiting the expression of the macrophage migration inhibitory factor (MIF)^17^. miR-19a suppresses HBP1 expression and up-regulates MIF to promote the release of inflammatory factors TNF-α and IL-6. In addition, miR-19a was up-regulated by the TNF-α in both THP-1 and RAW264.7 cells. These results imply that miR-19a is a pro-inflammatory miRNA during atherosclerosis.

## Results

### miR-19a is overexpressed in the plasma and atherosclerotic plaques of CAD patients and is induced by oxLDL in human macrophages

**Figure 1:**
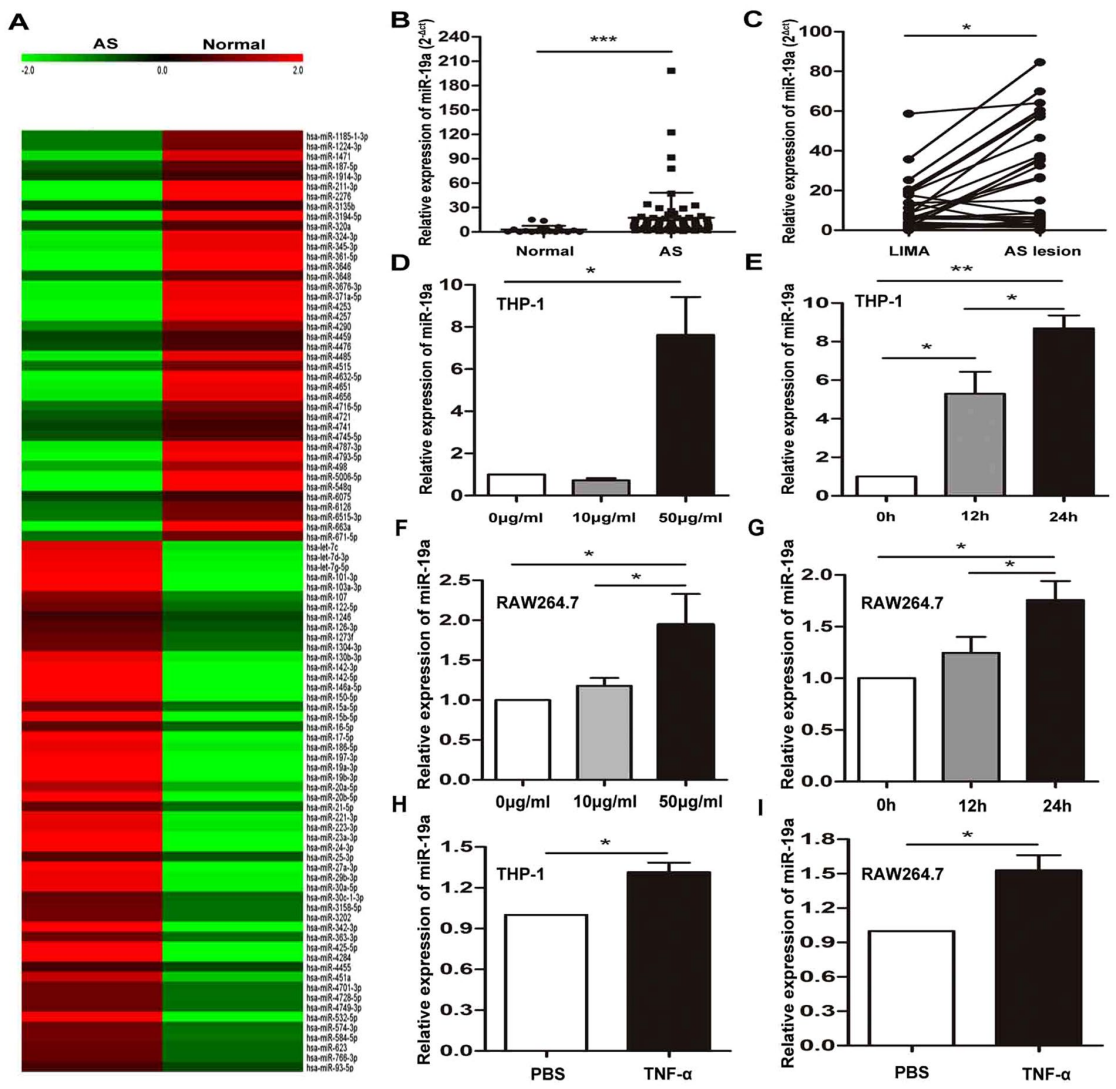
miR-19a is overexpressed in the plasma and atherosclerosis plaques of CAD patients and is induced by oxLDL in human macrophages. (**A**) The expression of miR-19a in a pool of plasma with CAD (n = 10) or normal control (n = 10) was determined by microarray. (**B**) The level of miR-19a in the plasma from patients with CAD (n = 66) and in normal control group (n = 18). Values were normalized to U6. In this dot plot, the horizontal line indicates the mean. (**C**) The level of miR-19a in the atherosclerotic lesion compared with the normal LIMA (n = 38). (**D**,**F**) qRT-PCR analysis of miR-19a expression in THP-1 cells and RAW264.7 cells. THP-1 cells were first stimulated with propylene glycol monomethyl ether acetate (PMA, 100 nM) to differentiate into macrophages. Both cell lines were treated with oxLDL at the indicated doses (0, 10, 50 μg/ml). (**E**,**G**) qRT-PCR analysis of miR-19a expression in THP-1 cells and RAW264.7 cells. Cells were treated with oxLDL (50 μg/ml) for the indicated times (0, 12, 24 h). (**H**,**I**) qRT-PCR analysis of miR-19a expression in THP-1 cells and RAW264.7 cells, which were treated with TNF-α (20 ng/ml). *P<0.05, **P<0.01, ***P<0.001. Results are presented as mean ± SD of 3 independent experiments as determined by Bonferroni’s multiple comparison test after one-way ANOVA or Student’s t test.

To determine whether miRNAs was involved in atherosclerosis, a microarray was used to analyze plasma of patients with CAD and healthy controls. The results showed that miR-19a levels were significantly higher in CAD patients (Fig. 1A). To validate that the expression of miR-19a was increased in patients with AS, we also measured the miR-19a expression level using qRT-PCR and found that miR-19a level was indeed higher in the plasma from patients with CAD (Fig. 1B). Next, we determined the relative expression level of miR-19a in 38 pairs of atherosclerotic lesion and normal LIMA from the same patients using qRT-PCR and found that the miR-19a level was remarkably higher in the atherosclerotic lesion than in the normal LIMA (Fig. 1C). To investigate the expression of miR-19a in an *in vitro* model, THP-1 cells were treated with 100 nM PMA and subsequently stimulated with oxLDL at different concentrations (0, 10, 50 μg/ml) for 24 h or with 50 μg/ml oxLDL for different durations (0, 12, and 24 h)^18,19^. We found that the expression of miR-19a is upregulated by oxLDL stimulation in a dose- and time-dependent manner (Fig. 1D,E). Furthermore, mouse RAW 264.7 cells was used to test whether miR-19a is regulated by oxLDL stimulation mice. We found that the expression of miR-19a was upregulated by oxLDL stimulation in a dose- and time-dependent manner (Fig. 1F,G). Interesting, miR-19a was also upregulated by the TNF-α in both THP-1 and RAW264.7 cells (Fig. 1H,I).

### Inflammatory cytokine production and lipid uptake is regulated by miR-19a

**Figure 2:**
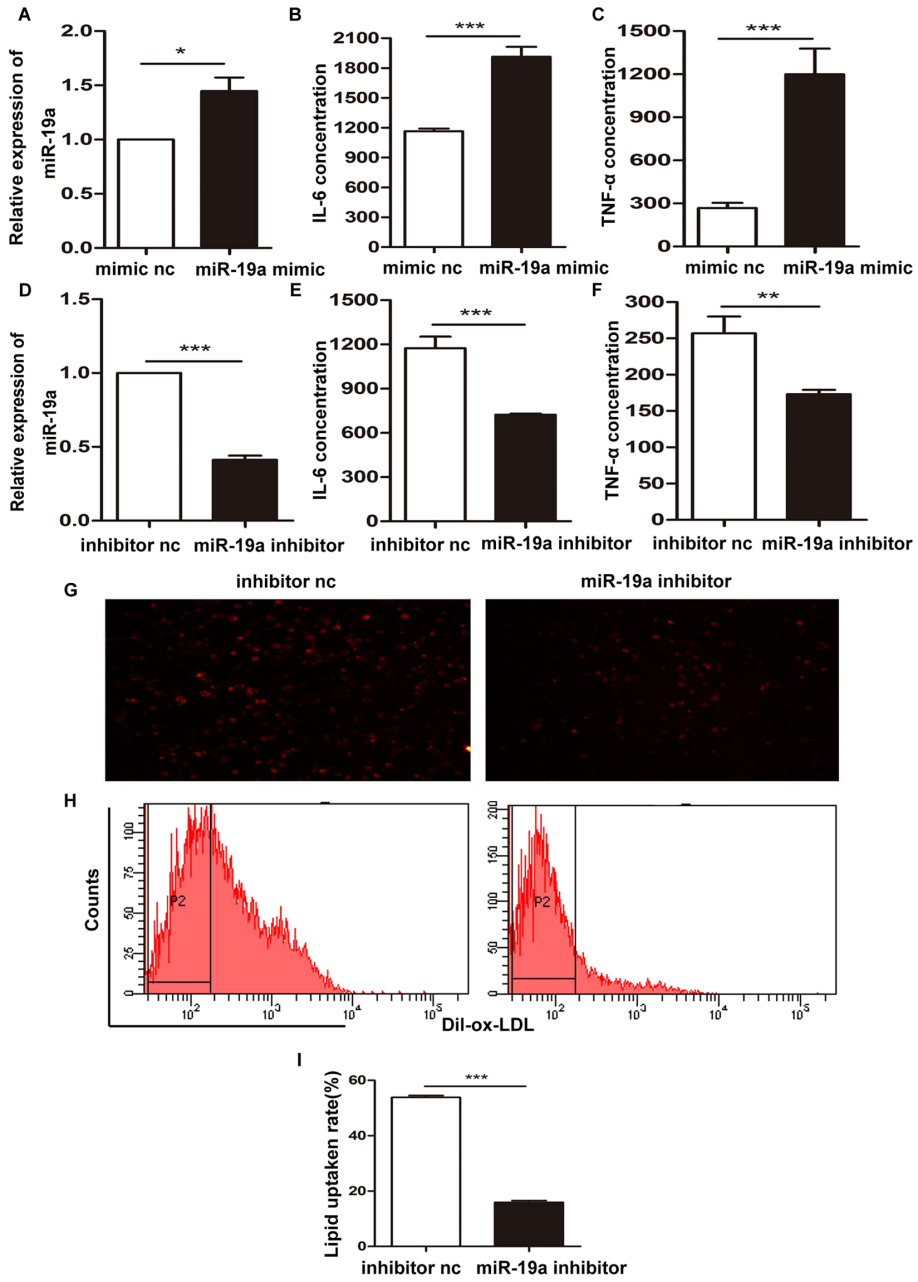
Inflammatory factor production and lipid uptake is regulated by miR-19a. (**A**,**D**) The expression of miR-19a was examined by qRT-PCR after transfection of miR-19a mimic, inhibitor, and control (100 nM) into macrophages that had been previously differentiated from THP-1 cells. (**B**,**E**) THP-1-derived macrophages were transfected with miR-19a mimic or its inhibitor at the indicated dose, the concentration of IL-6 in macrophages supernatant were measured by ELISA Kit. (**C**,**F**) THP-1-derived macrophages were transfected with miR-19a mimic or inhibitor at the indicated dose, the concentration of TNF-α in macrophages supernatant were measured by ELISA Kit. (**G**,**H**) THP-1-derived macrophages were transfected with miR-19a inhibitor at the indicated dose for 24h, and then incubated with Dil-oxLDL for 6h. Lipid uptake of macrophages was assessed by fluorescent imaging and fluorescence activated cell sorting (magnification 200×). (**I**) The quantitative results of the lipid uptake. *P<0.05, **P<0.01, ***P<0.001. Results are presented as mean ± SD of 3 independent experiments as determined by Student’s t test.

Inflammatory cytokine production and lipid uptake of macrophages play a crucial role in macrophage-derived foam cell formation^20^. THP-1-derived macrophages were transfected with miR-19a mimic or miR-19a inhibitor at the indicated dose (Fig. 2A,D). TNF-α and IL-6 concentration in macrophages supernatant were measured by ELISA Kits. We found that overexpression of miR-19a in macrophages enhanced IL-6 and TNF-α production (Fig. 2B,C). Conversely, inhibition of miR-19a had the opposite effects (Fig. 2E,F). Next, to investigate whether miR-19a regulates lipid uptake of macrophages, we transfected THP-1-derived macrophages with a miR-19a inhibitor, then incubated cells with Dil-oxLDL for 6 h. Both fluorescent imaging and fluorescence activated cell sorting (FACS) results showed that inhibition of miR-19a repressed the lipid uptake of macrophages and reduced foam cell formation (Fig. 2G,H and I).

### miR-19a directly targets the 3′UTR of HBP1

**Figure 3:**
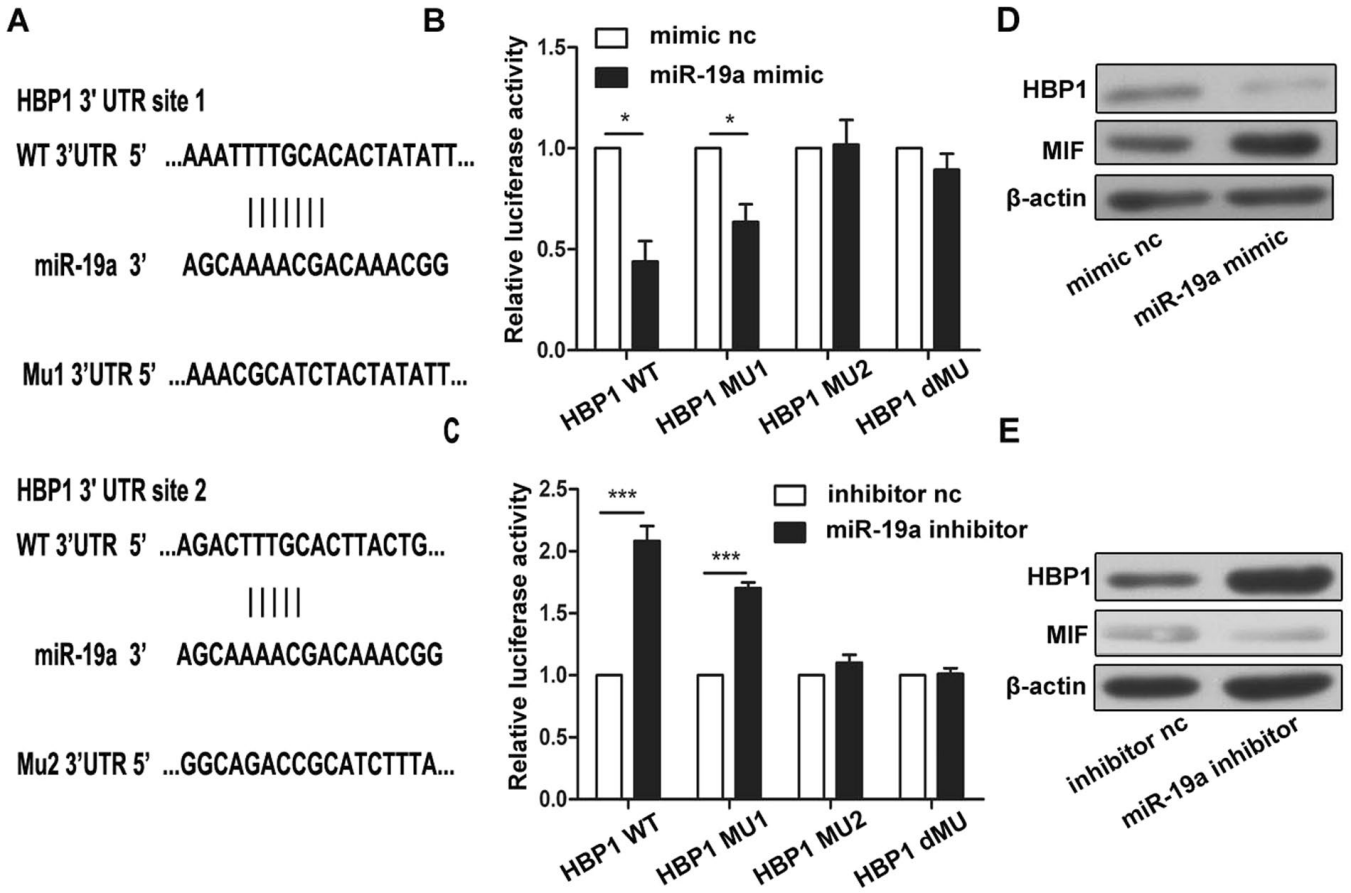
miR-19a directly targets the 3′UTR of HBP1. (**A**) The predicted binding site of miR-19a in the 3′-UTR of HBP1 is indicated. Two HBP1 3′-UTR mutants with mutation in the miR-19a binding site are also shown. (**B**,**C**) After co-transfection with the pRL-TK plasmid carrying a wild type or mutant 3′-UTR sequence and a miR-19a mimic (10 pmol) or a miR-19a inhibitor (10 pmol) into HEK-293T cells for 48 h, the luciferase activity was measured. (**D**,**E**) The protein level of HBP1 was measured by western blot after transfection with the miR-19a mimic (100 nM), the miR-9a inhibitor (100 nM), the mimic control or the inhibitor control into THP-1 cells at 48 h. *P<0.05, **P<0.01, ***P<0.001. Results are presented as mean ± SD of 3 independent experiments as determined by Student’s t test.

To search potential targets of miR-19a, we performed several miRNA target prediction algorithms available online, including TargetMiner, MirDB, PicTar, Targetscan. HBP1 (HMG box-transcription protein1), which acts as a transcriptional repressor, is identified as a potential target of miR-19a. There are two putative binding sites in the HBP1 3′UTR to the seed sequence of miR-19a and both are highly conserved in human, chimp, mouse, rat, and dog. We made mutants for both sites and performed a luciferase reporter assay in HEK-293T cells by transfecting a miR-19a mimic (or a miR-19a inhibitor) and a luciferase reporter downstream with either the wild type HBP1 3′UTR or its mutants (Fig. 3A). With the miR-19a mimic, we found that miR-19a suppressed reporter activity and that the site 2 mutant rescued such suppression, whereas the site 1 mutant had no effect (Fig. 3B). In addition, introduction of the miR-19a inhibitor caused an evidently increase in luciferase activity (Fig. 3C), which was abolished by the mutation at site 2 but not at site 1. Double mutations had similar impact as the site 2 mutant. We also directly assessed the expression of HBP1 and MIF (a gene regulated by HBP1). Overexpression of miR-19a decreased the level of HBP1 and increased the level of MIF; miR-19a inhibition had the opposite effects (Fig. 3D,E). All the results suggest that miR-19a regulate HBP1 by directly target the site 2 on the 3′UTR of HBP1.

### HBP1 as a functional target of miR-19a regulates the expression of MIF in foam cell formation

**Figure 4:**
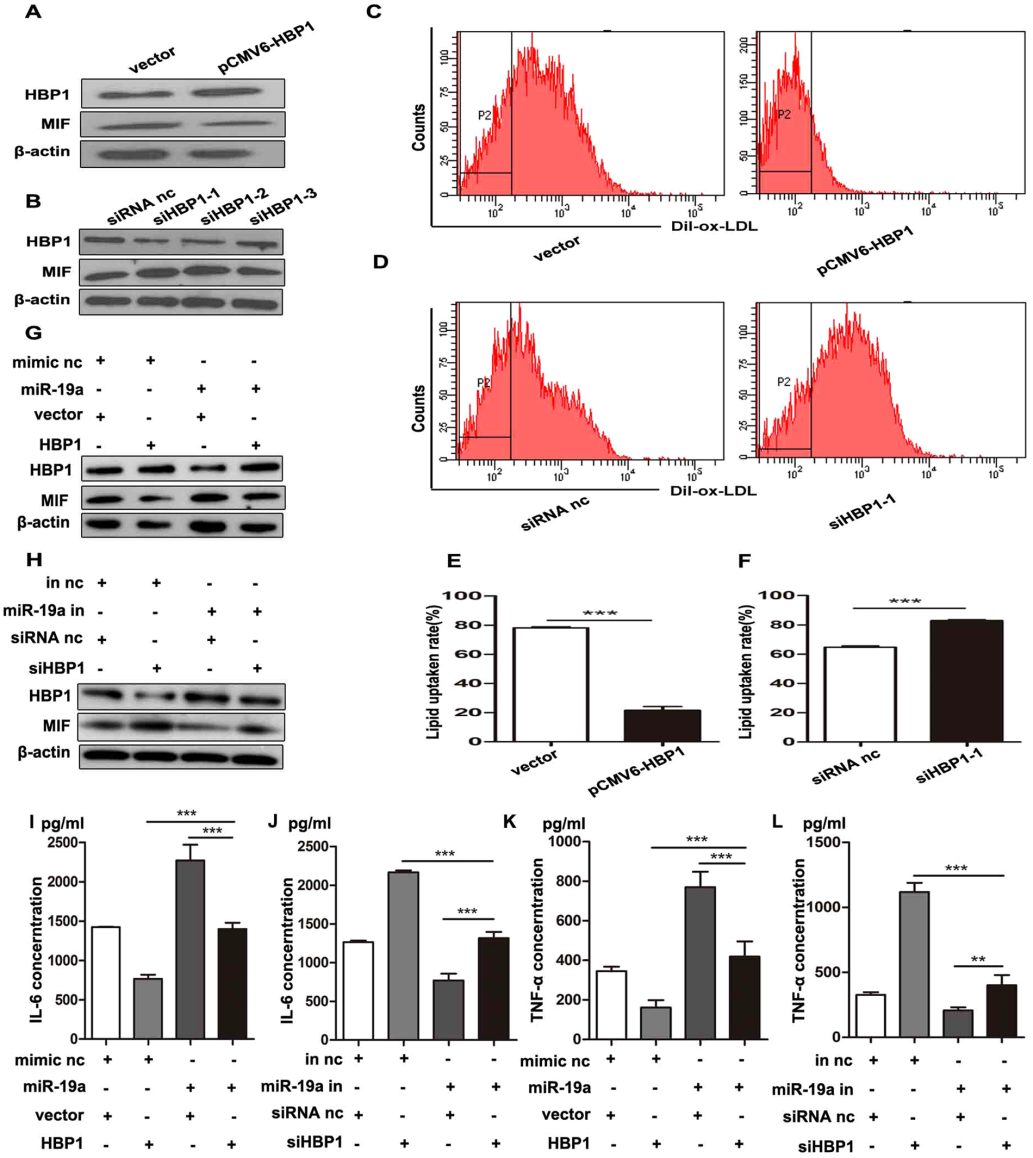
The role of HBP1 and MIF in foam cell formation. (**A**) Western blot analysis of the protein level of HBP1 to examine the efficiency of overexpression. The intracellular protein concentration of MIF was determined by western blot to determine the effect of HBP1 overexpression on MIF intracellular protein level, with β-actin as a control. Overexpression group was transfected with pCMV6-AC plasmid (Vector, 1 µg), pCMV6-AC-HBP1 plasmid (HBP1, 1 µg). (**B**) Western blot analysis of the protein level of HBP1 to examine the efficiency of knockdown. The intracellular protein concentration of MIF was determined by western blot to determine the effect of HBP1 knockdown on MIF intracellular protein level, with β-actin as a control. Knockdown group was transfected with 3 HBP1 siRNA (siHBP1–1, siHBP1–2, siHBP1–3, 50 pmol/ml), siRNA control (siRNA nc, 50 pmol/ml) for 24 h. (**C**,**D**) Lipid uptake of macrophages was assessed by and fluorescence activated cell sorting after HBP1 overexpression or knockdown. (**E**,**F**) The quantitative results of the lipid uptake after HBP1 overexpression or knockdown. (**G**) Western blot analysis to examine the protein levels of HBP1 and MIF after transfection with miR-19a mimic, pCMV6-AC-GFP (vector), or pCMV6-AC-HBP1 (HBP1) or co-transfection with miR-19a mimic and HBP1, with β-actin as a control. (**H**) Western blot analysis to examine the protein levels of HBP1 and MIF after transfection with miR-19a inhibitor, siRNA control, or siHBP1–1 or co-transfection with miR-19a inhibitor and siHBP1–1, with β-actin as acontrol. (**I**,**J**) The secreted protein concentration of IL-6 was determined by ELISA to determine the effects of HBP1 and miR-19a modulation. (**K**,**L**) The secreted protein concentration of TNF-α was determined by ELISA to determine the effects of HBP1 and miR-19a modulation. *P<0.05, **P<0.01, ***P<0.001. Results were presented as mean ± SD of 3 independent experiments as determined by Student’s t test.

To determine the role of HBP1 and MIF during foam cell formation, THP-1 derived macrophages were transfected with the HBP1 overexpression vector or 3 siRNAs against HBP1, named siHBP1–1, siHBP1–2, and siHBP1–3. Introduction of the HBP1 vector elevated the expression of HBP1, but reduced the expression of MIF in THP-1-derived macrophages (Fig. 4A). Three siRNAs knocked down the level of HBP1 and upregulated MIF (Fig. 4B). In addition, overexpression of HBP1 remarkably attenuated lipid uptake by macrophages (Fig. 4C,E), whereas HBP1 knockdown led to enhanced lipid uptake (Fig. 4D,F). These results suggest that HBP1 regulates MIF during foam cell formation.

To further examine whether HBP1 is a functional target of miR-19a during foam cell formation, THP-1 cells were transfected with the miR-19a mimic, siHBP1–1, or the HBP1 overexpression vector. We found that overexpression of HBP1 rescued HBP1 downregulation that was mediated by miR-19a (Fig. 4G) and weaken the pro-inflammatory effects (increased IL-6 and TNF-α secretion) caused by miR-19a (Fig. 4I,K). In contrast, inhibition of HBP1 by the siRNA restrained the protein level of HBP1 that was induced by knockdown of miR-19a (Fig. 4H) and reversed the phenotype (reduced IL-6 and TNF-α secretion) caused by miR-19a knockdown (Fig. 4J–L). Taken together, these data indicate that HBP1 is a functional target of miR-19a, which directly targets its 3′UTR, and that HBP1 serves as an effector of miR-19a by regulating MIF expression and IL-6 and TNF-α secretion and thereby affecting foam cell formation.

### AntgomiR-19a protects mice against atherosclerosis

**Figure 5:**
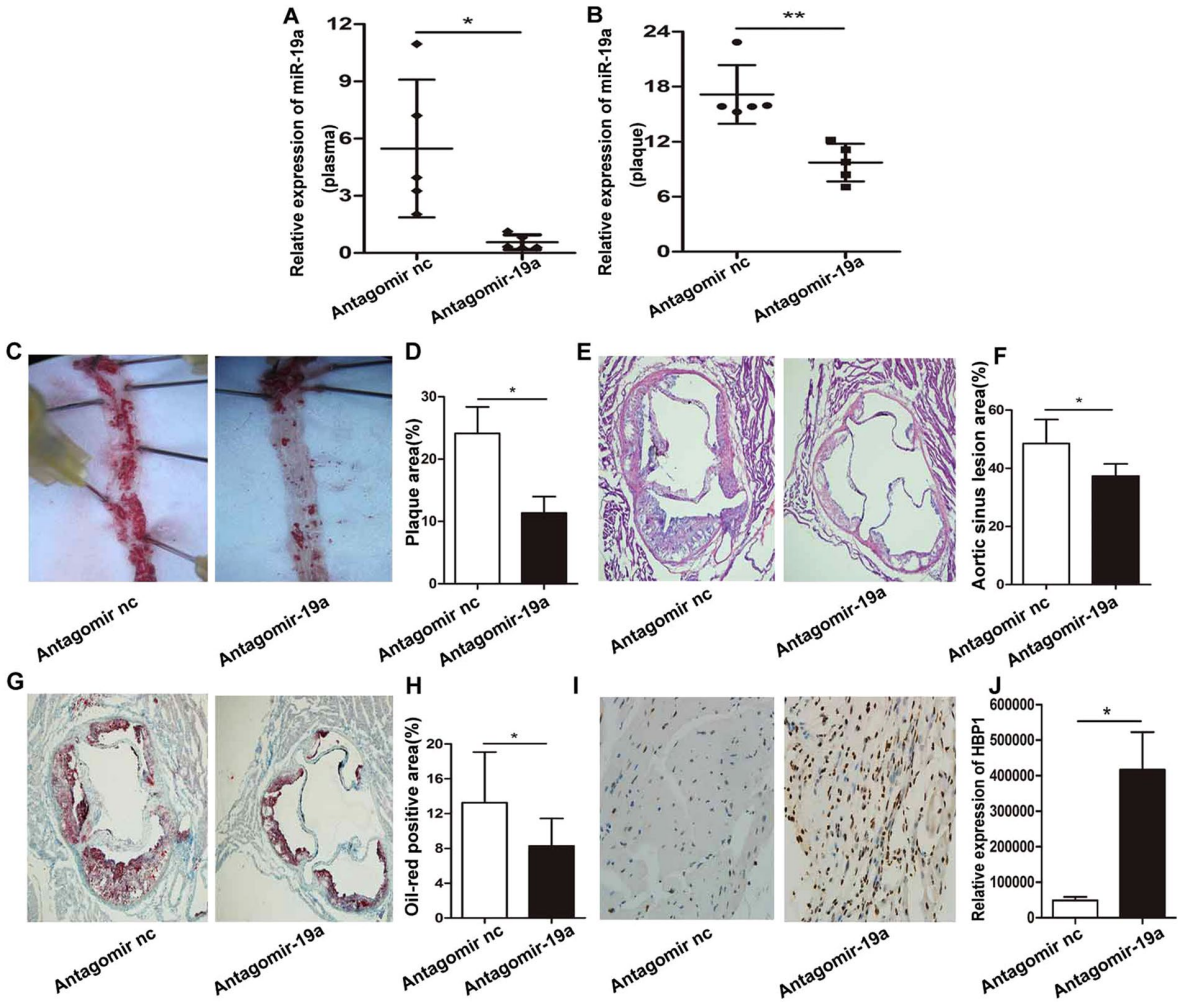
AntagomiR-19a protects mice against atherosclerosis. (**A**,**B**) The expression of miR-19a was determined by qRT-PCR in plasma and aortic tissue from ApoE^−/−^mice which were injected with antagomiR-19a or antagomiR control, n=5. (**C**,**D**) Atherosclerotic plaques was assessed by Oil Red O staining in the thoraco-abdominal aorta of ApoE^−/−^mice which were injected with antagomiR-19a or antagomiR control. The plaques area ware quantified by using imageJ. (**E**,**F**) Atherosclerotic plaques was assessed by representative hematoxylin-and eosin-stained at the aortic root sinus cryo-sections from antagomiR- and antagomiR-19a-injected ApoE^−/−^mice, and the plaques area ware quantified by using imageJ. (**G**,**H**) Atherosclerotic plaques was examined by representative Oil Red O staine at the aortic root sinus cryo-sections from antagomiR- and antagomiR-19a-injected ApoE^−/−^mice, and the plaques area ware quantified by using imageJ. (**I**,**J**) The expression of HBP1 in the arterial wall of ApoE-null mice treated by antagonistic miR-19a or the control was analyzed by immunohistochemistry. *P<0.05, **P<0.01, ***P<0.001. Results are presented as mean ± SD of 3 independent experiments as determined by Student’s t test.

Finally, we used an animal model to determine the role of miR-19a in atherosclerosis. Six-week-old male ApoE^−/−^ mice were fed with HFD^21^, and then injected with antagomiR-19a or control antagomiR via the tail vein. The expression level of miR-19a in plasma and aortic tissue from antagomiR-19a-injected ApoE^−/−^mice was decreased notably compared with the control group (Fig. 5A, B). Atherosclerotic lesions were examined by an *en face* analysis of the thoracoabdominal aorta and the cross-sections of the root of aorta. The atherosclerotic lesions throughout the aorta in ApoE^−/−^ mice were reduced in the antagmiR-19a group compare with the control group (Fig. 5C,D). The plaque area in the root of aorta were examined using H&E staining. We found that the size of the plaques in antagmiR-19a treated mice was reduced compare with the control (Fig. 5E,F). Furthermore, Oil Red O staining results showed that lipid loading in the plaques from antagmiR-19a-injected ApoE^−/−^ mice was decreased significantly (Fig. 5G,H). Additionally, we also detected significantly higher expression of HBP1 in the arterial wall of antagonistic miR-19a-treated ApoE null mice immunohistochemistry than the control, implying that miR-19a represses HBP1 expression in mice (Fig. 5I,J). Besides, there were no statistical differences in total triglycerides and cholesterol, LDL, and HDL in the plasma, body weight, or collagen contents of atherosclerotic plaques between the two groups. Overall, these results suggest that miR-19a inhibition attenuates atherosclerosis in mice.

## Discussion

**Figure 6:**
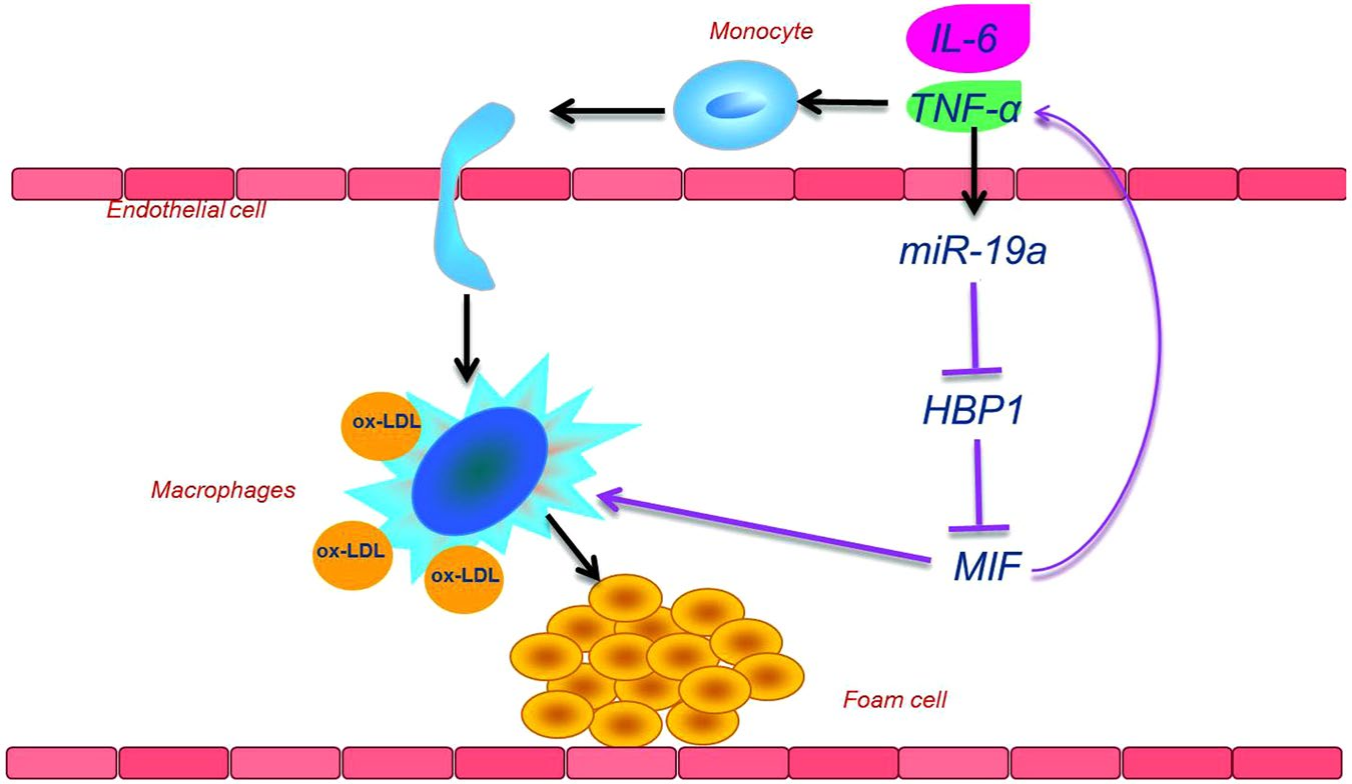
Schematic presentation of miR-19a in atherosclerosis. Activated endothelial cells recruit monocytes, which differentiate into macrophages when exposed to inflammation factors such as IL-6 and TNF-α. TNF-α-mediated expression of miR-19a upregulates MIF by targeting HBP1 and promotes the release of inflammatory cytokines TNF-α and IL-6. This TNF-α-miR-19a-HBP1-MIF forward feedback loop results in foam cell formation and promotes AS progression.

Atherosclerosis continues to be one of the biggest threat to human health and its initiation, prevention, and treatment invite extensive biological and medical research^22,23^. miR-19a is an important member of the miR-17–92 polycistronic gene cluster, which include seven microRNAs (miR−17–5p and −3p, miR-18a, miR19a and b, miR-20a and miR-92a)^24^. In this study, we show that 4 of them (17–5p, 19a, 19b, and 20a) are upregulated in the AS lesions when comparing with the normal control. This microRNA gene cluster could be activated during atherosclerosis to promote vascular inflammation and foam cell formation. We reveal enrichment of serum miR-19a in coronary patients in comparison with healthy individuals, and miR-19a overexpression in atherosclerotic tissues of arterial walls compared to LIMA tissues. Other reports suggest that miR-19a is up-regulated in endothelial blood vessel cells under hypoxia-inducible factor and shear stress^25–27^, and miR-19a suppresses IL-10 expression in peripheral B cells from patients with atherosclerosis^28^ suggesting that miR-19a may participate in atherosclerosis. It has been reported that HBP1 could be a target gene of miR-19a and miR-17–5p in tumor cells^29,30^. In the present work, we ascertain HBP1 as a novel target gene of miR-19a in atherosclerosis. Cytokines and inflammatory factors are important players in chronic AS inflammation^31–33^. HBP1, as a transcription repressor, specifically inhibit the expression of MIF^17^, a chemokine with pro-inflammatory and pro-atherogenic properties^34,35^. We show that the miR-19a down-regulates HBP1, resulting in MIF up-regulation and increased secretion of inflammatory cytokines TNF-α and IL-6, while miR-19a inhibition has the opposite effects. We also demonstrate that overexpression of miR-19a promotes foam cell formation. In addition, miR-19a is upregulated by TNF-α. Thus there is a forward feedback loop during atherosclerosis (Fig. 6): TNF-α stimulates the expression of miR-19a to suppress HBP1 and subsequently elevate MIF production, which in turn increases TNF-α secretion.

We also demonstrate the role of miR-19a *in vivo* using an animal model of atherosclerosis. High-fat fed ApoE^−/−^ mice are treated with a miR-19a antagonist, antagomiR-19a. The expression levels of miR-19a in the blood and arteries of the antagomiR-19a group are significantly lower than that in the control group, Additionally, the expression of HBP1 in the arterial wall of ApoE-null mice is upregulated with antagonistic miR-19a treatment. Histological analyses support that thoracic and abdominal aorta specimens have significantly fewer and smaller atherosclerotic plaques and reduced lipid contents in antagonist group compared with the control group. These results indicate that miR-19a inhibition alleviates the inflammatory response and slows the development of AS. MicroRNAs are involved in the regulation of blood lipids^36^; among them, miR-19b is reported to regulate HDL-C and LDL-C in the plasma of ApoE-null mice^37^. However, there are no statistical differences in total triglycerides, cholesterol, LDL, and HDL between the plasma of mice treated with antangomiR-19a and that of the control in our experiments. These results support differential roles of miR-19a and miR-19b in regulating circulating lipids, as miR-19b targets ABCA1 and miR-19a targets HBP1.

In summary, our present work demonstrates that miR-19a is elevated in the circulation and atherosclerotic lesions of patients with coronary artery disease. Through TNF-α-miR-19a-HBP1-MIF pathway, miR-19a up-regulates MIF and promotes the release of inflammatory cytokines TNF-α and IL-6. TNF-α promotes the expression of miR-19a, resulting in a forward feedback formed (Fig. 6). This study implies miR-19a as a promoter to inflammation and atherosclerosis. Inhibition of miR-19a, therefore, may be a novel strategy to combat atherosclerosis.

## Materials and Methods

### Patient characteristics

We enrolled 66 patients with CAD, who were diagnosed by clinical symptoms and coronary angiography from the Second XiangYa Hospital (Changsha, China) between March 2016 and August 2016. Eighteen healthy volunteers were recruited as the control group. We also identified 38 pairs of atherosclerotic lesions from aorta and normal left internal mammary artery (LIMA) from the same patients. Patients with the following disease were excluded: 1. any inflammatory disease that could activate monocyte such as pericarditis, pneumonia, bronchial asthma and infectious diseases caused by bacteria or virus; 2. severe diseases of cardiovascular system, such as acute heart failure or myocardial infarction. Informed consent was obtained from all subjects and this research conforms to the principles outlined in the Declaration of Helsinki. The human subject protocol was approved by the Medical Ethics Committee of the Second Xiangya Hospital.

### Plasma collection and storage

Peripheral blood from patients and control group were collected in EDTA tubes and processed within 2 hours by centrifuging at 1,000 g at 4 °C for 10 minutes. Plasma was transferred to a fresh RNase/DNase-free 1.5 ml EP tube (Axygen,CA,USA) and centrifuged at 16,000g at 4 °C for 10 minutes. The supernatant was transferred to another fresh RNase/DNase-free tubes and stored at −80 °C.

### Cell culture and establishing the foam cell model

THP-1 cells and HEK-293T cells were purchased from the American Type Culture Collection (ATCC). THP-1 cells were maintained in RPMI 1640 medium (Gibico, Carlsbad, CA, USA), HEK-293T cells were cultured in DMEM (Gibico, Carlsbad, CA, USA). Both media were supplied with 10% fetal bovine serum (Gibco, GrandIsland, NY, USA), 1% penicillin/streptomycin solution (Termo Fisher Scientifc, Rockford, IL, USA). Cells were cultured in a 5% CO_2_ incubator at 37 °C. To generate foam cells, THP-1 cells were seeded at 1×10^6^ per ml with 100 nM phorbol 12-myristate 13-acetate (PMA, Sigma-Aldrich, St. Louis, MO, USA) for 24 hours to differentiate into adherent macrophages, which were then stimulated with 50 μg/ml oxLDL (Luwen, Shanghai, China) for 24 hours to establish foam cells^38,39^.

### Measurement of Dil-oxLDL Uptake

The uptake of Dil-oxLDL, oxidized low density lipoprotein, labeled with 1,1′-dioctadecyl-3,3,3′,3′-indocarbocyanine perchlorate in macrophages was measured by Fluorescent microscope and FACS assay.THP-1-derived macrophages were transfected with miR-19a inhibitor for 48 hours, and then incubated with 20 μg/ml Dil-oxLDL (Yiyuan biotechnology Guangzhou, China) for 6 hours at 37 °C For FACS assay, cells were washed three times with PBS before subjected to FACS (fluorescence activated cell sorting).

### Western blot

Cells were lysed in RIPA buffer (Beyotime, Shanghai, China) and protein concentration was measured by the BCA method (Pierce, Rockford, IL, USA). Soluble lysate was mixed with loading buffer and boiled for 10 minutes before loaded onto a 10% SDS-PAGE and transferred to PVDF membranes (Bio-Rad, Hercules, CA, USA). Antibodies against HBP1 (1:1000, Abcam, Cambridge, MA, USA) and MIF (1:500, Abcam, Cambridge, MA, USA) were purchased from Abcam, β-Actin (Santa Cruz, Delaware, CA, USA) was used as a control. Bound antibodies were detected with secondary HRP-conjugated antibodies (1:40000, Santa Cruz, Delaware, CA, USA) and visualized by ECL chemiluminescent substrate (GE, Buckinghamshire, UK).

### RNA isolation and qRT-PCR

Total RNA from THP-1 macrophages and atherosclerotic lesions was isolated using TRIzol reagent (Invitrogen, Vilnius, Lithuania). qRT-PCR was measured using SYBR Green (Applied Biosystems, Carlsbad, CA, USA) in an ABI StepOne Plus qPCR instrument. Plasma miRNA from CAD patients and healthy volunteers were extracted by the mirVana PARIS kit (Ambion, Carlsbad, CA, USA) according to the manufacturer’s instructions. miRNA reverse transcription was performed using the Taqman MicroRNA Reverse Transcription kit (Applied Biosystems, Carlsbad, CA, USA). All PCR results were normalized using U6 as a reference and expressed as 2^−∆∆ct^.

### ELISA

Secreted pro-inflammatory factors TNF-α and IL-6 concentration in THP-1 mediated macrophages supernatant was measured by respective ELISA kits (R&D Systems, Minneapolis, MN, USA) according to the manufacturer’s instructions.

### Luciferase reporter assay

1×10^4^ HEK-293T cells were seeded into 96-well plates for 24 hours, and the cells were transfected with pRL-TK-HBP1–3′UTR or pRL-TK-HBP1–3′UTR mutant (mut1 or mut2) vectors (100 ng), 10 ng of pGL3 control (Promega, Madison, USA), and 10 pmol miR-19a mimic or miR-19a inhibitor using Lipofectamine LTX and Plus reagent (Invitrogen, Carlsbad, CA, USA). Luciferase activity was measured using the Dual-Glo luciferase report assay system (Promega, Madison, USA) after 48 hours using the manufacturer’s instructions.

### Animal experiments

Six-week old male *ApoE* ^−/−^mice (C57BL/6J) were purchased from HFK bioscience company (Beijing, China) were maintained at 22±2 °C, relative humidity 55%±5% with a 12hours light/dark cycle. After fed with a rodent chow diet (4.5%fat) for a week, *ApoE* ^−/−^ mice were fed with western diet (21% fat, 1.25% cholesterol; HFK bioscience) for 12 weeks. Mice were then randomized into 2 groups (n=6 mice, respectively): antagomiR-19a-injected and control antagomiR-injected group. The mice received tail vein injections of 25mg/kg antagomiR-19a (GenePharma, Shanghai, China) or antagomiR once per week for 4 weeks^40,41^. After fasting for 6h, mice were euthanized and blood samples from the retro-orbital plexus, heart and aorta were collected for further analyses. All animal experiments were carried out in accordance with the protocol approved by the Institutional Animal Care and Use Committee of the Central South University.

### Statistical analysis

The data were presented as the mean±SEM; Student’s t-test was carried out for comparison between two groups and one-way analysis of variance (ANOVA) among multiple groups using GraphPad Prism software. Differences with P values of<0.05 are considered significant.

## Electronic supplementary material

Supplementary materials
