# Supplementary material for: MircroRNA-19a promotes vascular inflammation and foam cell formation by targeting HBP-1 in atherogenesis
Source: Sci Rep. 2017 Sep 21;7:12089. doi: 10.1038/s41598-017-12167-z (PMC5608705; doi:10.1038/s41598-017-12167-z)
Supplement: Supplementary file 1 — Supplementary materials [file 41598_2017_12167_MOESM1_ESM.pdf]

MircroRNA-19a promotes vascular inflammation and foam cell formation by targeting *HBP-1* in atherogenesis

Heming Chen[1], Xiaoyi Li[2], Shuiyi Liu[2], Lu Gu[1], Xinmin Zhou[1]

1. Department of Cardiovascular Surgery, Second XiangYa Hospital, Central South University, Changsha, 410011, China.

2. Department of Medical Laboratory, Central Hospital of Wuhan, Wuhan 430014, China.

Supplementary materials

Supplementary Figure S1：Full-length blots for Figure 3D

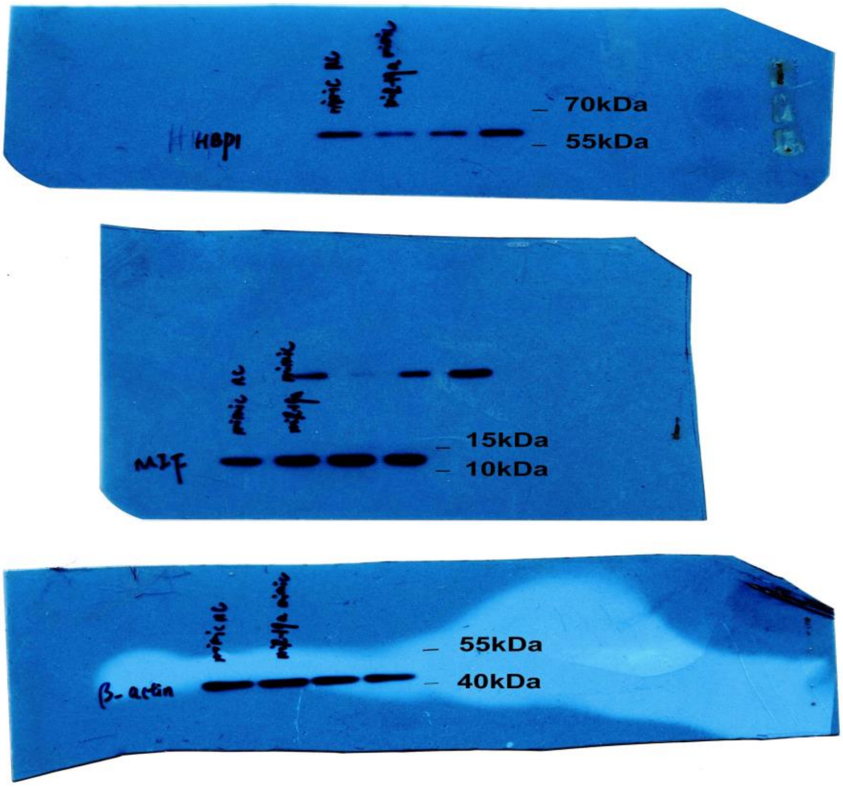

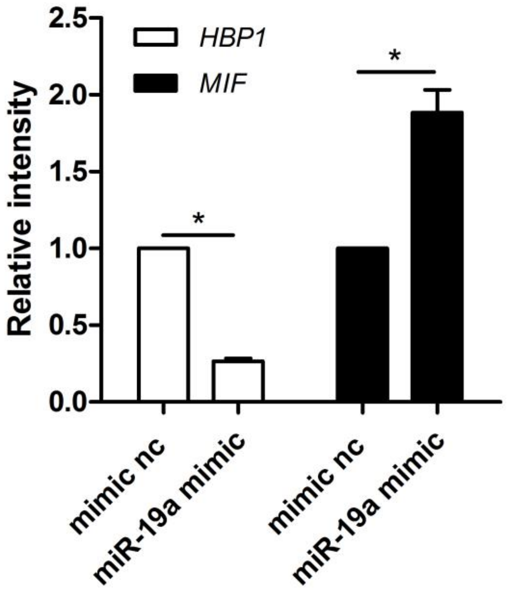

Supplementary Figure S2: Full-length blots for Figure 3E

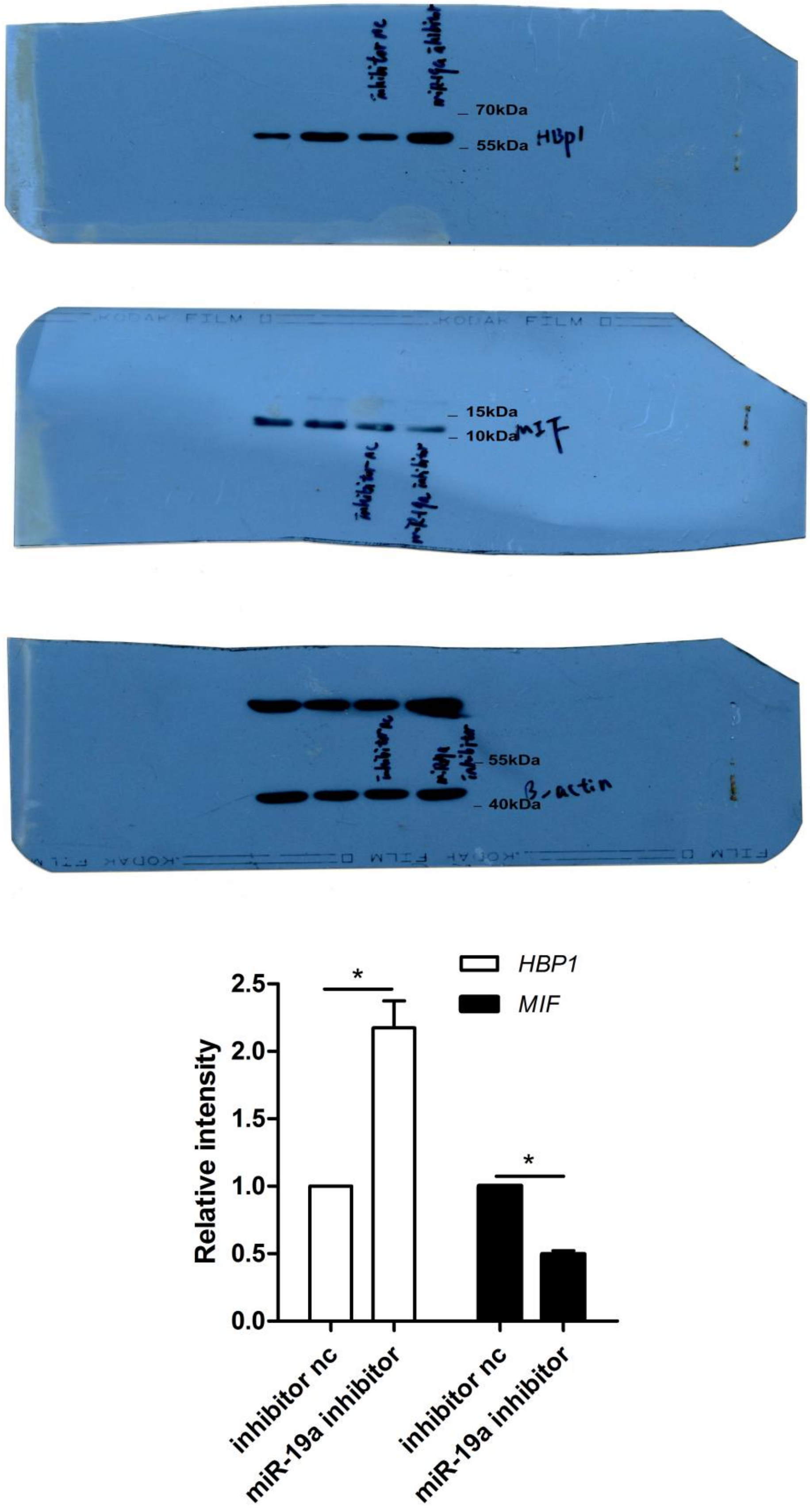

Supplementary Figure S3：Full-length blots for Figure 4A

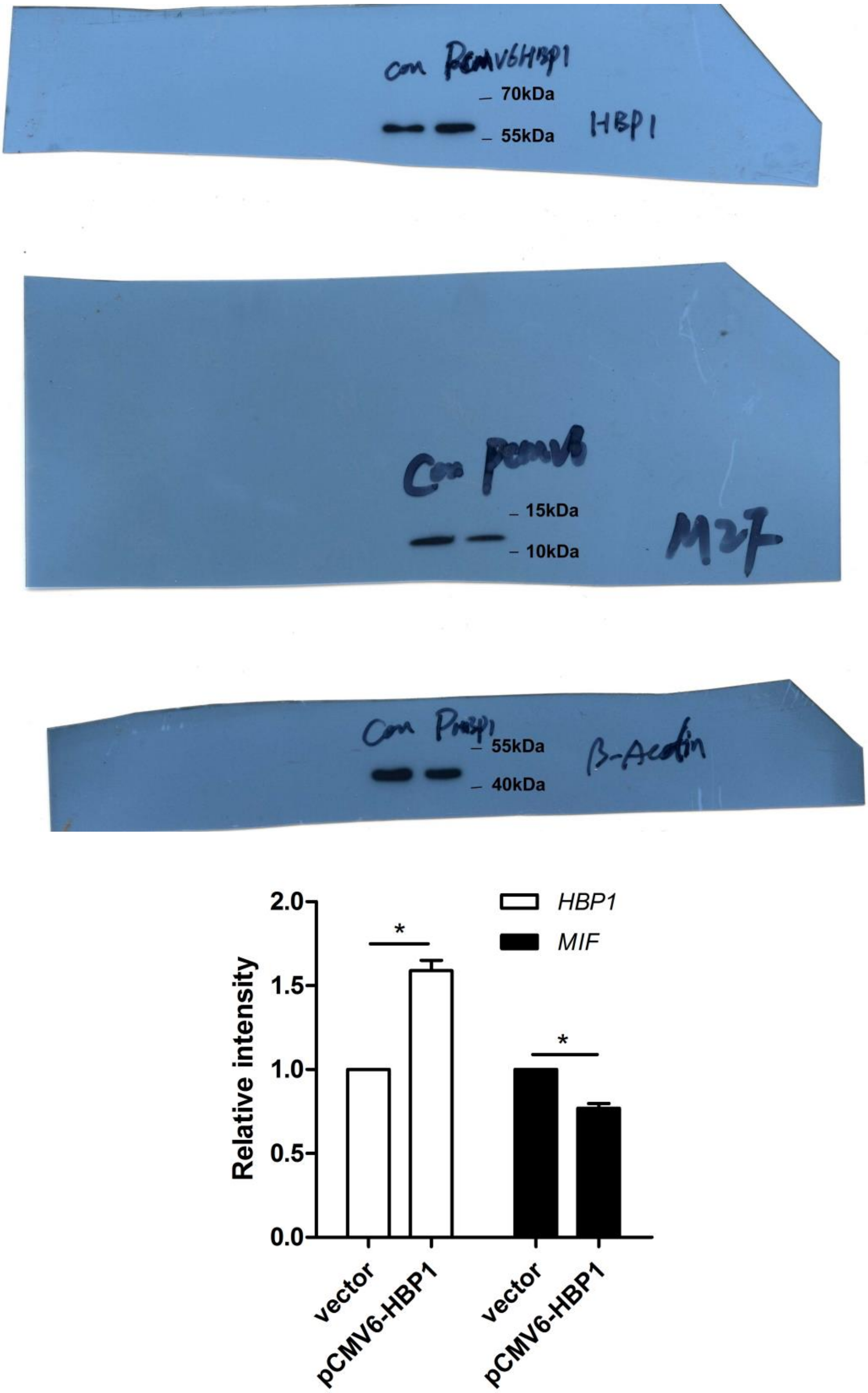

Supplementary Figure S4: Full-length blots for Figure 4B

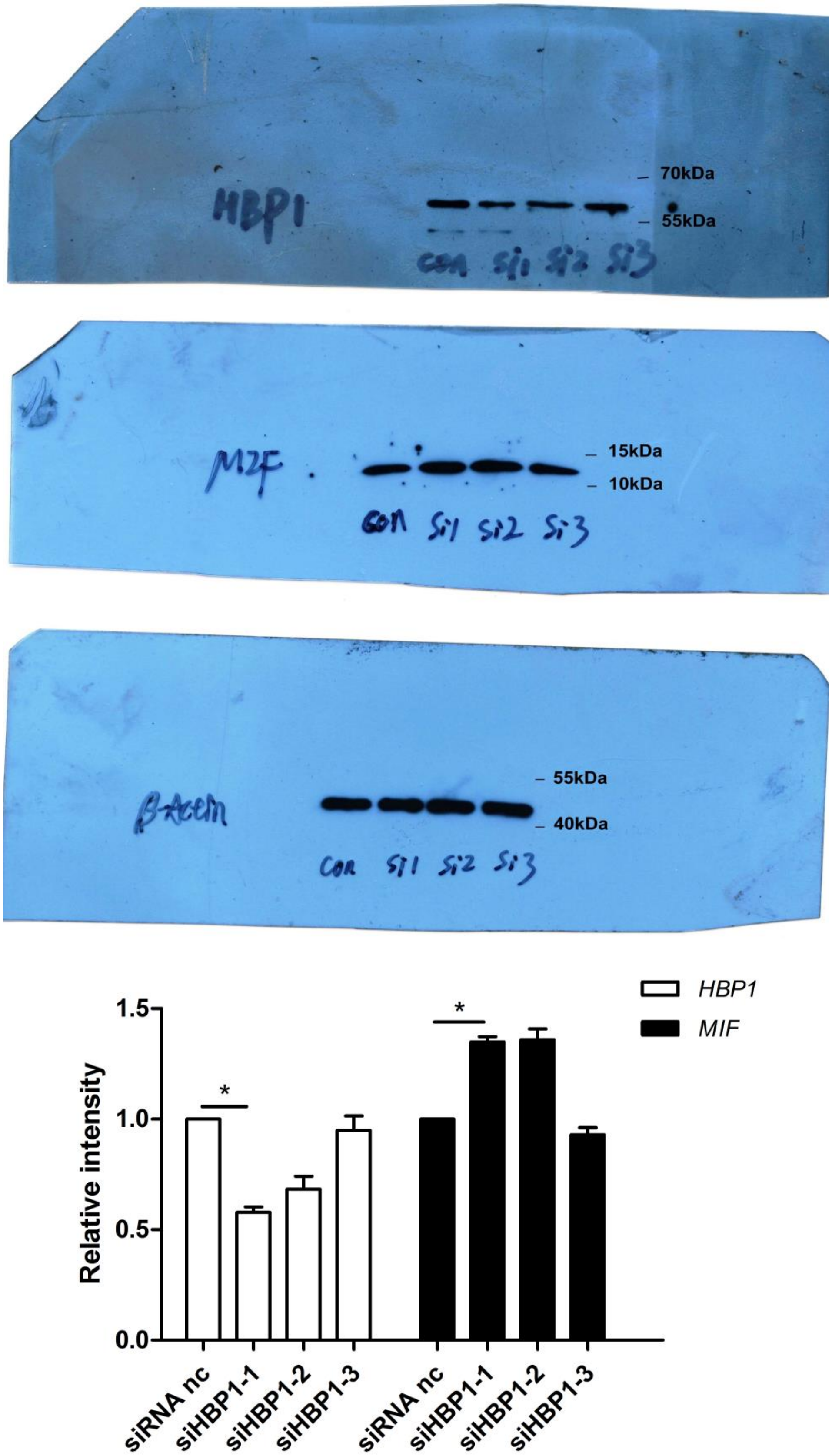

Supplementary Figure S5: Full-length blots for Figure 4G

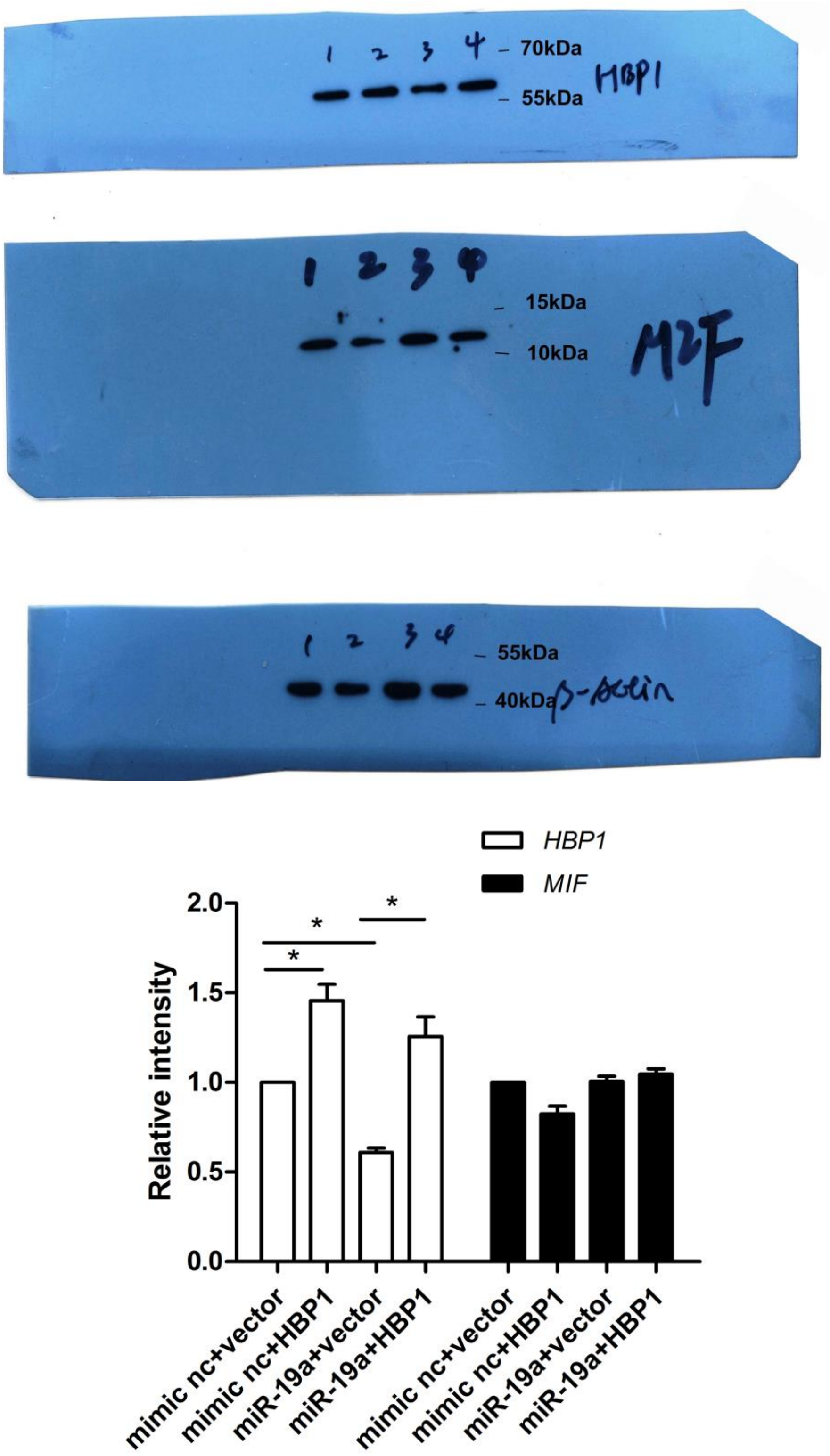

Supplementary Figure S6： Full-length gels for Figure 4H

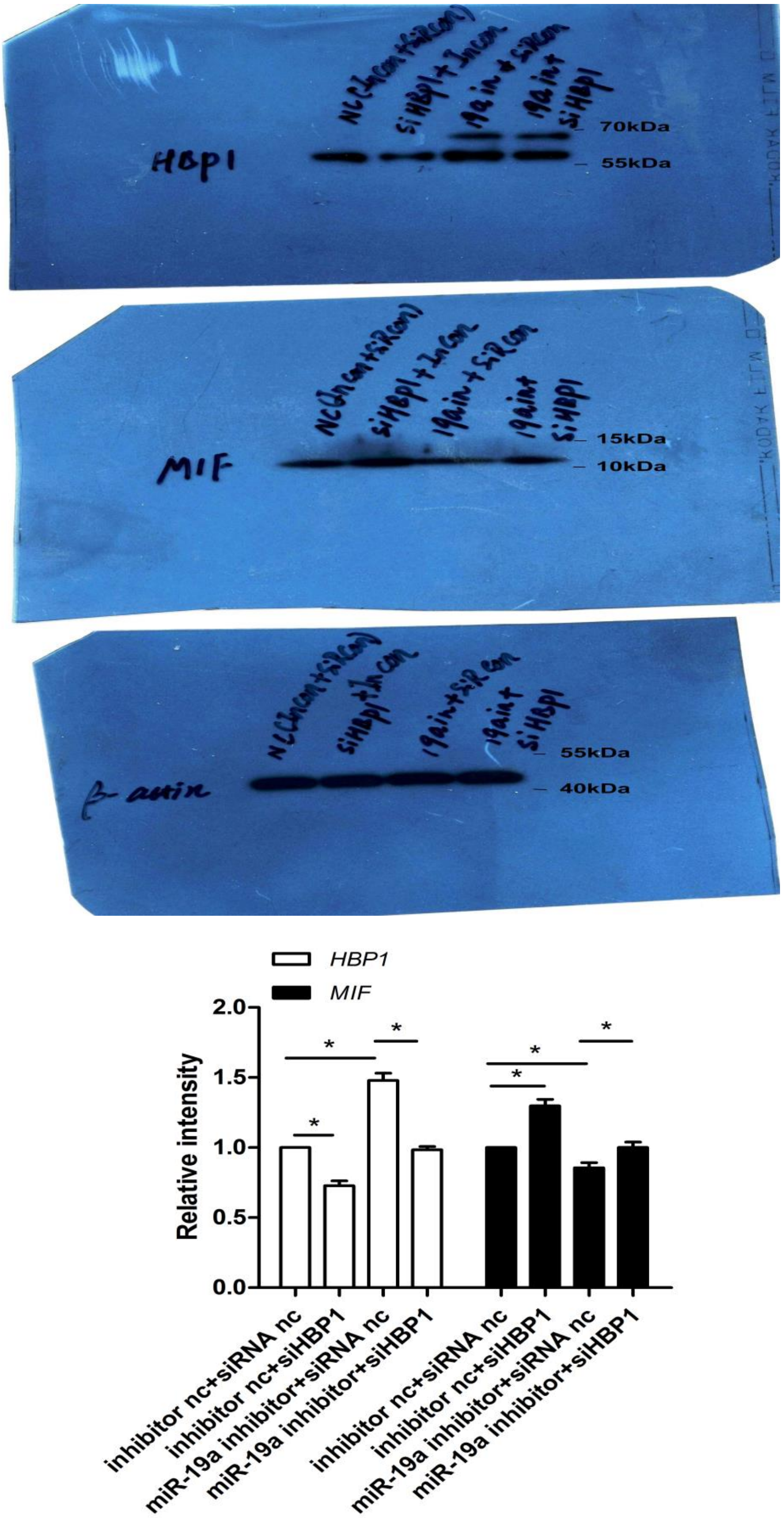

Supplementary Figure S7: The conservation of the binding site of miR-19a in HBP1 Figure S7. The nucleotides complementary to the miR-19a seed sequences are highly conserved in human, mouse, rat, rabbit and cow.

miR-19a-3p	5’-U**GUGCAAA**UCUAUGCAAAACUGA-3’
Hsa	3’-A**CACGUUU**UAAAUCCCCGUUGUA-5’
Mmu	3’- A**CACGUUU**UAACUCCCCGUUAUA-5’
Rno	3’- A**CACGUUU**UAACUCCCCGUUAUA-5’
Ocu	3’- A**CACGUUU**UAACUCCCCGUUGUA-5’
Bta	3’- A**CACGUUU**UAAAUCCCCGUUGUA-5’
